# Melanins Protect *Sporothrix brasiliensis* and *Sporothrix schenckii* from the Antifungal Effects of Terbinafine

**DOI:** 10.1371/journal.pone.0152796

**Published:** 2016-03-31

**Authors:** Rodrigo Almeida-Paes, Maria Helena Galdino Figueiredo-Carvalho, Fábio Brito-Santos, Fernando Almeida-Silva, Manoel Marques Evangelista Oliveira, Rosely Maria Zancopé-Oliveira

**Affiliations:** Laboratório de Micologia, Instituto Nacional de Infectologia Evandro Chagas, Fundação Oswaldo Cruz, Rio de Janeiro, Brazil; Instituto de Salud Carlos III, SPAIN

## Abstract

Terbinafine is a recommended therapeutic alternative for patients with sporotrichosis who cannot use itraconazole due to drug interactions or side effects. Melanins are involved in resistance to antifungal drugs and *Sporothrix* species produce three different types of melanin. Therefore, in this study we evaluated whether *Sporothrix* melanins impact the efficacy of antifungal drugs. Minimal inhibitory concentrations (MIC) and minimal fungicidal concentrations (MFC) of two *Sporothrix brasiliensis* and four *Sporothrix schenckii* strains grown in the presence of the melanin precursors L-DOPA and L-tyrosine were similar to the MIC determined by the CLSI standard protocol for *S*. *schenckii* susceptibility to amphotericin B, ketoconazole, itraconazole or terbinafine. When MICs were determined in the presence of inhibitors to three pathways of melanin synthesis, we observed, in four strains, an increase in terbinafine susceptibility in the presence of tricyclazole, a DHN-melanin inhibitor. In addition, one *S*. *schenckii* strain grown in the presence of L-DOPA had a higher MFC value when compared to the control. Growth curves in presence of 2×MIC concentrations of terbinafine showed that pyomelanin and, to a lesser extent, eumelanin were able to protect the fungi against the fungicidal effect of this antifungal drug. Our results suggest that melanin protects the major pathogenic species of the *Sporothrix* complex from the effects of terbinafine and that the development of new antifungal drugs targeting melanin synthesis may improve sporotrichosis therapies.

## Introduction

Sporotrichosis is a subcutaneous mycosis that has areas of high endemicity in Latin America, especially in Brazil [1], and Asia, mainly in China [2]. Sporotrichosis is caused by members of the *Sporothrix* complex, of which *Sporothrix brasiliensis*, *Sporothrix globosa*, and *Sporothrix schenckii* are the species most frequently associated with human disease [3]. Sporadic cases are caused by *Sporothrix mexicana* [4], *Sporothrix luriei* [5], and *Sporothrix pallida*, formerly *Sporothrix albicans* [6].

Several clinical manifestations are observed in sporotrichosis, ranging from cutaneous localized disease to life-threatening, disseminated opportunistic infections in immunosuppressed patients [1]. Spontaneous cures are uncommon [7] and the majority of patients require antifungal therapy. Itraconazole is the drug of choice for sporotrichosis treatment [8, 9]. Terbinafine is an alternative therapeutic for patients that cannot use itraconazole due to drug interactions or side effects, and it has an efficacy similar to itraconazole [10]. Amphotericin B is used in severe cases of infection [9], and ketoconazole has been successfully used in the treatment of feline sporotrichosis [11], but is not frequently used due to toxicity issues. Potassium iodide can be used for this mycosis [12], but its use is limited due to side effects [13].

The pathogenic *Sporothrix* species have the ability to produce pigmented colonies through the production of melanin [14]. Three different types of melanin can be produced by these fungi, with DHN-melanin being the main pigment produced by these fungi [15]. DHN-melanin is constitutively produced by *Sporothrix* conidia under several environmental, laboratory, and parasitic conditions, and the synthesis of this polymer can be blocked by specific inhibitors [14]. The assembly of DHN-melanin does not require the presence of specific precursors in the environment, since it is produced via acetyl-coA originated from glycolysis [16]. In contrast, eumelanin and pyomelanin are additionally produced if precursors such as 3, 4-dihydroxy-L-phenylalanine (L-DOPA) or L-tyrosine, respectively, are available during fungal growth [16–18]. It is important to note that these different melanins can be produced concurrently if the specific inductors are present. *In vivo* production of melanin by members of the *Sporothrix* complex is supported by the isolation of melanin particles (‘ghosts’) from the tissues of infected hamsters and by the detection of antibodies against DHN- and eumelanin in the sera from patients with different manifestations of sporotrichosis [16, 19]. Melanins have been shown to be important virulence factors in several fungi, including protecting fungi from the harsh conditions found during parasitism due to the immune response of the host [20]. Another important function of fungal melanins is the protection against antifungal drugs [21]. *Histoplasma capsulatum*, *Cryptococcus neoformans*, and *Paracoccidioides brasiliensis* demonstrate increase resistance to amphotericin B and caspofungin if they grow under melanin-inducing conditions [21, 22]. Our group also described that pyomelanin and eumelanin can temporarily protect *S*. *brasiliensis* from the fungicidal effects of amphotericin B [17]. Here we investigate whether the three different melanin types expressed by *S*. *schenckii* and *S*. *brasiliensis* have a role in protection against the major antifungal drugs historically used in the treatment of sporotrichosis.

## Material and Methods

### Antifungal drugs

Amphotericin B, itraconazole, ketoconazole, and terbinafine were purchased from Sigma-Aldrich (St Louis, MO). Serial dilutions of drugs were performed in dimetilsulfoxide (DMSO) in order to obtain working concentrations ranging from 0.015 to 8 mg/L, unless otherwise described.

### Strains and culture conditions

Two *S*. *brasiliensis* (CBS120339 and IPEC26449) and four *S*. *schenckii* strains (IPEC23252, IPEC24372, ATCC16345, and ATCC32286) were used throughout the study. They comprise the *S*. *brasiliensis* type strain, a clinical *S*. *brasiliensis* strains, two clinical *S*. *schenckii* strains from different geographic origins (Brazilian states of Espírito Santo and Rio de Janeiro) and two reference *S*. *schenckii* strains. *Aspergillus flavus* ATCC204304 and *Aspergillus fumigatus* ATCC204305 were used as controls in microdilution assays. Strains were grown on potato dextrose agar (PDA), the culture medium recommended by the CLSI for fungal growth during inoculum preparation [23], at room temperature, a condition in which only DHN-melanin is produced [15, 16]. For eumelanin and pyomelanin production, L-DOPA 1mM or L-tyrosine 10 mM, respectively, were added to the culture medium. These concentrations were previously determined as optimal for eumelanin and pyomelanin production in *Sporothrix spp*. [14, 16]. Under these conditions, eumelanin or pyomelanin, respectively, are produced in addition to DHN-melanin.

### Evaluation of melanin production

The *Sporothrix* strains were cultured on the above-described agars for 7 days prior to use in the antifungal tests. In addition, the three types of melanin were checked on cells grown on RPMI1640 medium buffered at pH 7.0 with MOPS (3-[N-morpholino] propanesulfonic acid) at a concentration of 0.165 mol/L. DHN-melanin production was demonstrated by subjecting seven-day colonies pigmented colonies to a hot-acid treatment to isolate melanin particles of similar size and shape of the *Sporothrix* propagules [16]. Since DHN-melanin is not produced in mycelia, the presence of eumelanin was assessed through the isolation of melanin ‘ghosts’ of the same size and shape of *Sporothrix* hyphae [16]. The production of pyomelanin, the only soluble type of melanin produced by *Sporothrix* spp., was demonstrated by the detection of a soluble black pigment in culture supernatants that precipitates after hydrochloric acid treatment as previously described [17].

### Quantification of melanins

DHN- and eumelanin were semiquantitatively measured through visual inspection of the cultures as described previously [16] prior to the antifungal assays. Moreover, these measurements were further evaluated by reading the optical density of inoculums at 310 nm. The absorbance at 340 nm, the wavelength of maximum absorbance for *Sporothrix* pyomelanin [17], of culture supernatants was used to quantify pyomelanin.

### Antifungal susceptibility of melanized *Sporothrix* strains

The susceptibilities of *Sporothrix* cells grown for 7 days on potato dextrose agar (PDA) as recommended [23] with or without supplementation with L-DOPA or L-tyrosine were compared. A broth microdilution reference method for determining minimal inhibitory concentration (MIC) determinations was performed according to the CLSI standard protocol M38-A2 [23] using cells diluted to concentrations of 5×10^4^ CFU/mL. Wells containing RPMI-1640 medium with 1% DMSO were used as controls. Although this is a protocol for susceptibility testing of filamentous fungi, this protocol is currently indicated for *S*. *schenckii*, despite the fact that this is not the parasitic form of the fungus. Moreover, the reference method for broth dilution antifungal susceptibility testing of yeasts states that the method is not validated for the yeast forms of dimorphic fungus [24]. Plates were kept at 37°C in the dark and read after 50 hours. Three independent experiments were performed. Essential agreement between different MIC determinations of a strain grown under different conditions was defined if a MIC variation within two-fold dilution of the antifungal drug was observed [25–27].

### Antifungal susceptibility in presence of melanin inhibitors

The broth microdilution reference method was also performed using plates containing the melanin inhibitors tricyclazole (8 mg/L), glyphosate (100 mg/L), or sulcotrione (16 mg/L), as previously described [14, 16]. These inhibitors were added to wells concurrently with the fungal inocula, since there is no information regarding the degradation of these compounds in the above conditions. When appropriate, the antifungal concentration range were decreased from 8–0.015 mg/L to 1.0–0.002 mg/L to better evaluate MIC reductions in the presence of melanin inhibitors. Three independent experiments were performed.

### Minimal fungicidal concentration

The minimal fungicidal concentrations (MFC) of the tested antifungal drugs with or without the addition of chemical melanin inhibitors were determined. The microplates were vortexed and 10 μL from the wells were inoculated onto Sabouraud dextrose agar (Difco Laboratories, Sparks, MD) and incubated at 30°C for 7 days. Plates were assessed for identification of antifungal dilutions promoting more than 99% of cell death, i.e. growth of less than five colonies under these conditions, which correspond to 1% survival of the original inoculum. The lowest antifungal concentration fitting this description was considered the MFC. Three independent experiments were performed.

### Time-kill assays

The strains cultivated with or without L-DOPA or L-tyrosine were assessed for time killing by a 2×MIC concentration of terbinafine. Inocula were obtained from 7-day old cultures prepared in phosphate buffered saline (PBS) in a concentration of 1×10^5^ *Sporothrix* cells/mL with or without terbinafine and this suspension was incubated at 37°C. Cell viability was determined through a colony forming assay with 10-fold dilutions of the PBS suspensions of *Sporothrix* cells at 0, 2, 4, 6, 8, and 24 hours. The control values to calculate the percent survival are those obtained with fungal cells grown in the absence of terbinafine. This experiment was also performed with yeast-cells of two strains (*S*. *brasiliensis* IPEC 26449 and *S*. *schenckii* ATCC 16345) grown in brain heart infusion (BHI) medium at the same conditions described above, both in regular and 5%CO_2_ atmospheres, to verify if the protective role of *Sporothrix* melanins also apply to the parasitic morphology of these two species. Three independent experiments were performed.

### Checkerboard assay

To understand the effects of tricyclazole on terbinafine susceptibility, dilutions of these two chemicals were prepared in RPMI1640 buffered with MOPS pH7.0. The dilution range was 0.12 to 8 μg/mL for tricyclazole and 0.002 to 1 μg/mL for terbinafine. Plates were prepared mixing 50 μL of each concentration of tricyclazole with 50 μL of terbinafine in 96-well plates to obtain an 8-by-11 checkerboard design [28]. A fractional inhibitory concentration index (FICI) was determined as described [29] and interpreted as follows: synergy was considered if FICI≤0.5, antagonism was considered if FICI>4.0, and no interaction was considered when FICI were within the range 0.5–4.0 [30].

### Statistical analyses

The GraphPad Prism 5.0 software was applied to perform the data analysis. The non-parametric Wilcoxon test was used to compare survival of cells grown with different substrates. *P* values less than 0.05 were considered to be statistically significant.

## Results

### Melanin production under antifungal susceptibility testing conditions

**Fig 1:**
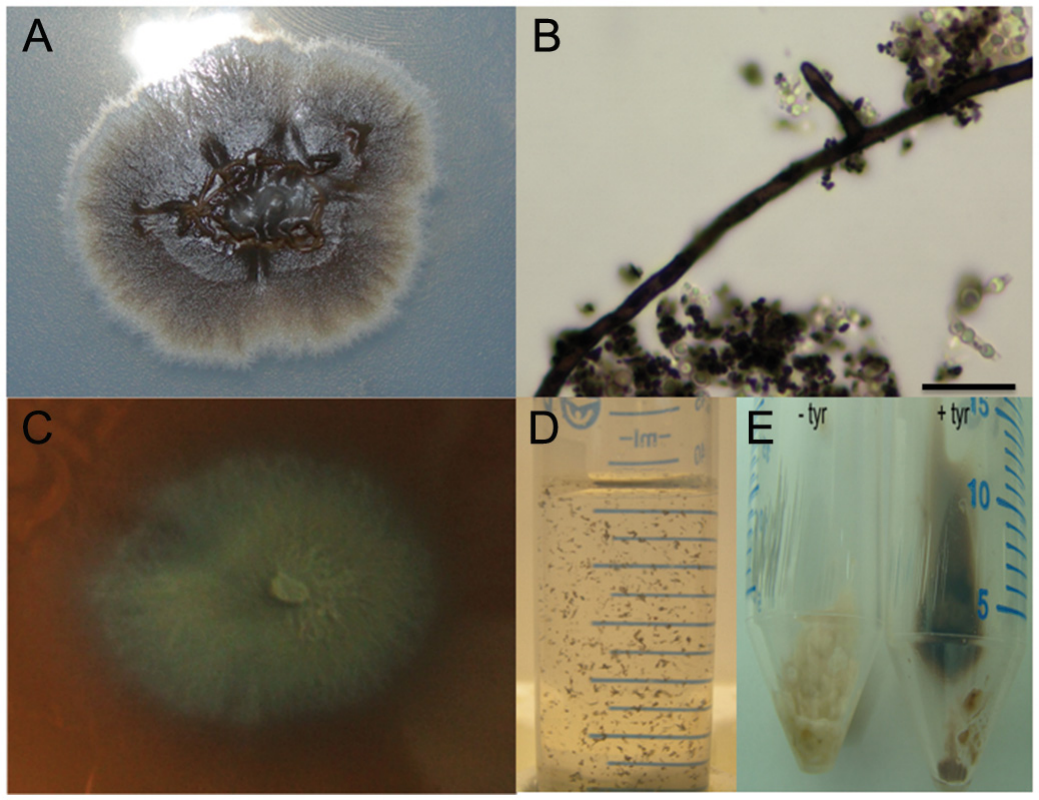
Production of melanin by *Sporothrix* under the conditions required for antifungal susceptibility tests. Data presented is for *S*. *brasiliensis* IPEC 26449, which is representative of all strains examined. (A) Dark pigmented colony on PDA after 7 days of incubation at 37°C; (B) Melanin ghosts from cells isolated from a seven day old culture in PDA supplemented with L-DOPA at 37°C showing similar structures in shapes and sizes relative to the *Sporothrix* hyphal and conidial propagules, compatible with eumelanin; (C) Dark pigment formation on PDA supplemented with L-tyrosine after 7 days of incubation at 37°C, compatible with pyomelanin; (D) Suspension of melanin ghosts isolated from a seven day old culture in RPMI1640 at 37°C; (E) *Sporothrix* cells harvested from RPMI1640 without L-tyrosine, at left, or supplemented with L-tyrosine, at right.

Each of the six strains included in this work produced the three different types of melanin by seven days of incubation at 37°C in PDA when supplemented with the specific inductors for eu- and pyomelanin (Fig 1A–1C). These strains were also able to yield melanin ghosts in the same shape and size of *Sporothrix* spp. conidia after hot-acid treatment when grown in RPMI-1640 medium without melanin precursors, i.e. L-DOPA or L-tyrosine (Fig 1D). Interestingly, the addition of L-DOPA to RPMI-1640 impaired fungal growth; hence, we were not able to isolate melanin particles using this medium. Pyomelanin was produced during fungal growth in RPMI-1640 medium supplemented with L-tyrosine (Fig 1E); however, pigmentation was visibly present only after nine days of growth. Quantification of melanins in cells recovered upon treatment with antifungal drugs was similar among the tested strains (*P*>0.05, OD at 310 nm range: 1.778–1.973). After treatment with the melanin inhibitors, OD at 310 nm of *Sporothrix* cells were close to the cell-free control (range: 0.113–0.130).

### Eumelanin and pyomelanin do not interfere significantly with *Sporothrix* spp. MICs

*Sporothrix* spp. cells grown in the presence of L-DOPA or L-tyrosine were incubated with the different antifungal dilutions and their MICs were compared to the MIC obtained according to the CLSI protocol [23]. Table 1 presents the MIC data for these conditions. We observed an essential agreement between all MIC readings, most of them within a one-fold dilution MIC difference, independent of the melanization status of the strains. A two-fold dilution MIC difference was observed for ketoconazole susceptibility of one *S*. *brasiliensis* and one *S*. *schenckii* strain grown in presence of L-tyrosine. However, it is interesting to note that the *Sporothrix* growth in the presence of L-tyrosine consistently decreases the MIC of ketoconazole, itraconazole, and terbinafine.

**Table 1:** Minimal inhibitory concentration (mg/L) of *Sporothrix* spp. strains grown in presence or absence of L-DOPA or L-tyrosine.

Strain	Amphotericin B			Itraconazole			Ketoconazole			Terbinafine		
	PDA^a^	LD^b^	Tyr^c^	PDA	LD	Tyr	PDA	LD	Tyr	PDA	LD	Tyr
*S*. *brasiliensis*												
CBS 120339	2.0	4.0	2.0	1.0	1.0	0.5	0.5	0.5	0.12*	0.06	0.03	0.03
IPEC 26449	1.0	1.0	2.0	1.0	1.0	0.5	0.5	0.5	0.25	0.03	0.015	0.015
*S*. *schenckii*												
IPEC 24372	8.0	4.0	4.0	1.0	1.0	1.0	0.5	0.5	0.25	0.03	0.03	0.015
IPEC 23250	8.0	4.0	4.0	1.0	1.0	0.5	0.5	0.5	0.12*	0.03	0.03	0.015
ATCC 16345	4.0	2.0	4.0	8.0	8.0	4.0	1.0	2.0	1.0	0.12	0.12	0.12
ATCC 32268	8.0	4.0	4.0	1.0	1.0	1.0	0.5	1.0	0.5	0.06	0.12	0.06

^a^ PDA, potato dextrose agar

^b^ LD, PDA supplemented with 1 mM L-DOPA

^c^ Tyr, PDA supplemented with 10 mM L-tyrosine

*indicates a greater than one fold dilution difference in MIC

### Tricyclazole increases *Sporothrix* susceptibility to terbinafine

*Sporothrix* spp. cells grown in PDA medium according to the CLSI standard protocol were inoculated into RPMI-1640 medium supplemented with one of the DHN, eumelanin, and pyomelanin inhibitors tricyclazole, glyphosate, or sulcotrione, respectively. Table 2 presents MIC values for the six strains in the presence and absence of these melanin inhibitors. Variations in MICs were generally within a one-fold dilution range comparing cells cultivated in PDA to those subjected to inhibitor treatment. For *S*. *schenckii* ATCC 16345, the MIC for terbinafine in PDA cultivated cells was 0.12 mg/L whereas treatment with tricyclazole reduced the MIC to 0.015 mg/L. Two-fold dilution difference in terbinafine MICs also occurred between control and tricyclazole treatment for both *S*. *brasiliensis* strains and *S*. *schenckii* ATCC 32268. Additionally, tricyclazole treatment reduced the MIC of ketoconazole in *S*. *brasiliensis* CBS 120339 compared to controls by two dilutions. Since all strains cultivated with tricyclazole yielded a MIC to terbinafine of 0.015 mg/L, the lowest detectable MIC according to the dilutions used in the experiments, we repeated the experiment with lower concentrations of terbinafine. A MIC of 0.008 mg/L was found for strain IPEC 26449. All other strains retained the MIC of 0.015 mg/L observed previously. Glyphosate and sulcotrione did not significantly interfere with MIC values of the strains for the four tested antifungal drugs.

**Table 2:** Minimal inhibitory concentrations (mg/L) of *Sporothrix* spp. strains in presence of the melanin inhibitors tricyclazole, glyphosate, and sulcotrione.

Strain	Amphotericin B				Itraconazole				Ketoconazole				Terbinafine			
	PDA	Tri^a^	Gly^b^	Sul^c^	PDA	Tri	Gly	Sul	PDA	Tri	Gly	Sul	PDA	Tri	Gly	Sul
*S*. *brasiliensis*																
CBS 120339	2.0	2.0	2.0	2.0	1.0	0.5	1.0	1.0	0.5	0.12*	0.5	0.5	0.06	0.015*	0.06	0.06
IPEC 26449	1.0	2.0	1.0	1.0	1.0	0.5	1.0	1.0	0.5	0.25	0.5	0.5	0.03	0.008*	0.015	0.015
*S*. *schenckii*																
IPEC 24372	8.0	4.0	4.0	4.0	1.0	1.0	1.0	1.0	0.5	0.25	0.5	0.25	0.03	0.015	0.03	0.03
IPEC 23250	8.0	4.0	2.0	2.0	1.0	0.5	0.5	0.5	0.5	0.25	1.0	0.25	0.03	0.015	0.03	0.03
ATCC 16345	4.0	4.0	2.0	2.0	8.0	4.0	2.0*	2.0*	1.0	1.0	2.0	2.0	0.12	0.015*	0.06	0.06
ATCC 32268	8.0	4.0	4.0	4.0	1.0	1.0	0.5	0.5	0.5	0.5	0.5	0.5	0.06	0.015*	0.06	0.03

^a^ Tri, RPMI-1640 medium with the antifungal drugs supplemented with tricyclazole

^b^ Gly, RPMI-1640 medium with the antifungal drugs supplemented with glyphosate

^c^ Sul, RPMI-1640 medium with the antifungal drugs supplemented with sulcotrione

*indicates a greater than one fold dilution difference in MIC between the different inhibitor conditions.

### Eumelanin influences the minimal fungicidal concentration of terbinafine

*Sporothrix* cells submitted to decreasing antifungal concentrations were evaluated by a plate colony count assay to define cell viability. Table 3 presents MFC values for the six strains against the four analyzed antifungal drugs. In all but one case, MFC values were similar for inocula prepared with cells expressing only DHN-melanin, DHN-melanin plus eumelanin, or DHN-melanin plus pyomelanin, with MIC varying by a one-dilution range. However, the MFC of *S*. *schenckii* IPEC 24372 from medium supplemented with L-DOPA was three dilutions greater than cells grown in PDA alone.

**Table 3:** Minimal fungicidal concentrations (mg/L) of *Sporothrix* spp. strains grown in presence or absence of L-DOPA or L-tyrosine.

Strain	Amphotericin B			Itraconazole			Ketoconazole			Terbinafine		
	PDA^a^	LD^b^	Tyr^c^	PDA	LD	Tyr	PDA	LD	Tyr	PDA	LD	Tyr
*S*. *brasiliensis*												
CBS 120339	8.0	4.0	4.0	4.0	2.0	4.0	4.0	4.0	4.0	0.12	0.12	0.12
IPEC 26449	8.0	4.0	4.0	8.0	8.0	4.0	4.0	4.0	4.0	4.0	2.0	4.0
*S*. *schenckii*												
IPEC 24372	8.0	4.0	8.0	8.0	8.0	8.0	2.0	2.0	2.0	0.5	4.0#	1.0
IPEC 23250	8.0	4.0	8.0	8.0	8.0	8.0	8.0	8.0	8.0	0.06	0.06	0.06
ATCC 16345	8.0	8.0	8.0	8.0	8.0	8.0	8.0	8.0	8.0	2.0	1.0	1.0
ATCC 32268	8.0	8.0	8.0	8.0	8.0	8.0	4.0	4.0	4.0	0.25	0.5	0.25

^a^ PDA, potato dextrose agar

^b^ LD, PDA supplemented with 1 mM L-DOPA

^c^ Tyr, PDA supplemented with 10 mM L-tyrosine

^#^greater than one-dilution increase compared to PDA cultivated cells

### Terbinafine fungicidal properties in presence of melanin inhibitors

*Sporothrix* cells were submitted to inhibitors of each of the three melanin biosynthesis pathways using different antifungal concentrations. Table 4 demonstrates that the MFCs were largely unchanged in the presence of these inhibitors, with specific exceptions. For *S*. *brasiliensis* IPEC 26449, sulcotrione significantly enhanced the killing effects of terbinafine and, to a lesser extent, glyphosate.

**Table 4:** Minimal fungicidal concentrations (mg/L) of *Sporothrix* spp. strains to four different antifungal drugs in presence of the melanin inhibitors tricyclazole, glyphosate, or sulcotrione.

Strain	Amphotericin B				Itraconazole				Ketoconazole				Terbinafine			
	PDA	Tri^a^	Gly^b^	Sul^c^	PDA	Tri	Gly	Sul	PDA	Tri	Gly	Sul	PDA	Tri	Gly	Sul
*S*. *brasiliensis*																
CBS 120339	8.0	2.0*	2.0*	2.0*	4.0	1.0*	8.0	8.0	4.0	2.0	8.0	2.0	0.12	0.12	0.12	0.12
IPEC 26449	8.0	4.0	2.0*	4.0	8.0	8.0	8.0	8.0	4.0	4.0	4.0	4.0	4.0	2.0	1.0*	0.12*
*S*. *schenckii*																
IPEC 24372	8.0	8.0	8.0	4.0	8.0	8.0	8.0	8.0	2.0	8.0	8.0	8.0	0.5	2.0#	1.0	0.5
IPEC 23250	8.0	4.0	8.0	2.0*	8.0	8.0	8.0	8.0	8.0	8.0	8.0	4.0	0.06	0.12	0.06	0.06
ATCC 16345	8.0	8.0	8.0	8.0	8.0	8.0	8.0	8.0	8.0	8.0	8.0	8.0	2.0	0.5*	1.0	1.0
ATCC 32268	8.0	8.0	4.0	8.0	8.0	8.0	8.0	8.0	4.0	8.0	8.0	4.0	0.25	1.0#	0.25	0.25

^a^ Tri, RPMI-1640 medium with the antifungal drugs supplemented with tricyclazole

^b^ Gly, RPMI-1640 medium with the antifungal drugs supplemented with glyphosate

^c^ Sul, RPMI-1640 medium with the antifungal drugs supplemented with sulcotrione

*greater than one-dilution reduction compared to PDA cultivated cells

^#^greater than a one dilution increase compared to PDA cultivated cells

### Eumelanin and pyomelanin protect *Sporothrix* spp. against terbinafine

**Fig 2:**
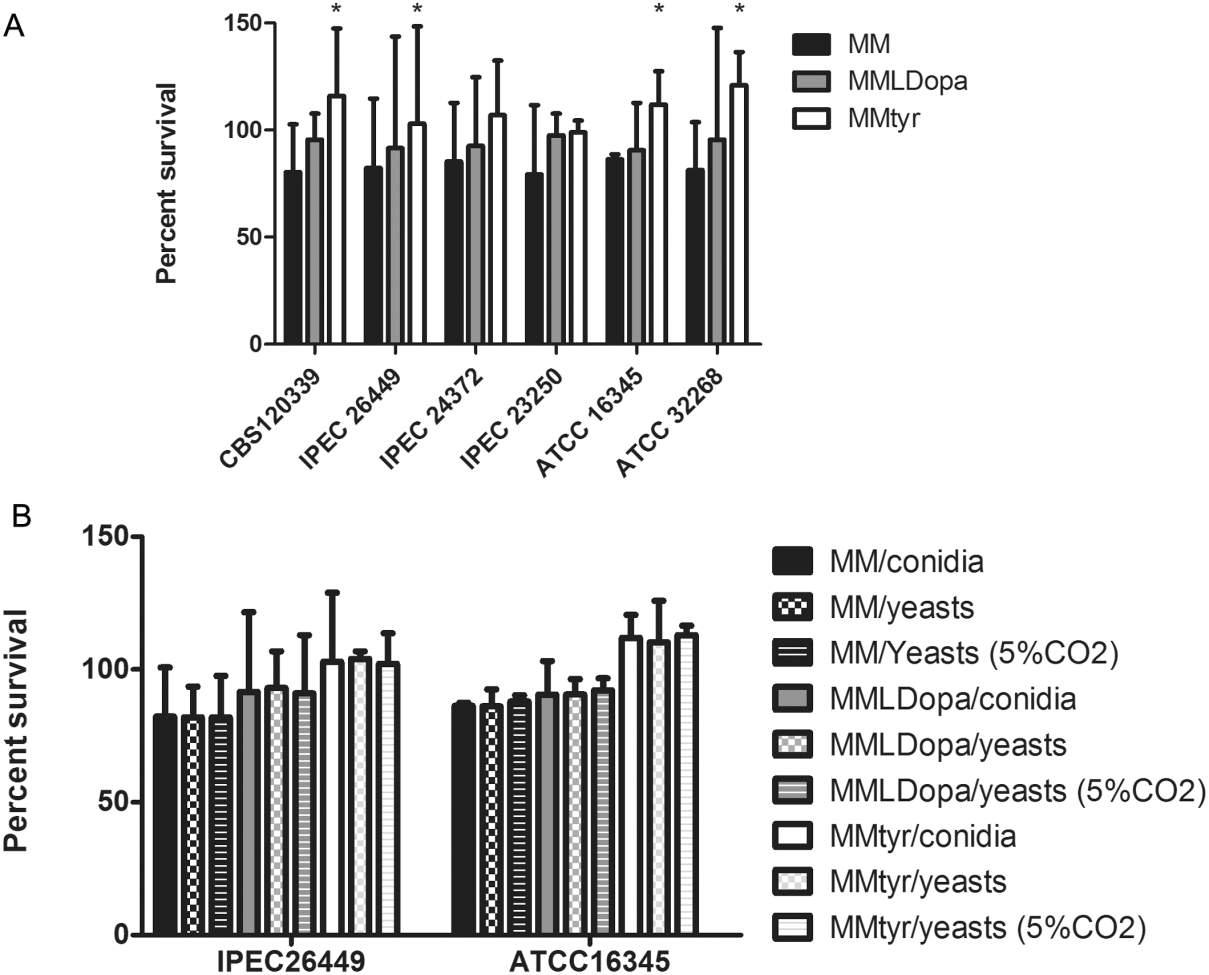
Protective role of *Sporothrix* melanins against terbinafine. (A) Percent survival of six *Sporothrix* spp. strains cultivated in minimal medium (MM), minimal medium with L-Dopa (MMLDopa), or minimal medium with L-tyrosine (MMtyr) after 24 hours of incubation with a two times MIC concentration of terbinafine. (B) Comparison between mycelia and yeast. The percent survival was calculated using *Sporothrix* cells grown without terbinafine as a control. Bars represent mean + standard deviation. * *P*<0.05.

Since resistance to the fungicidal effects of terbinafine by the microdilution assay was identified, we performed a time-kill experiment of terbinafine against the six strains under different melanization conditions. No differences in *Sporothrix* growth were observed at 2, 4, 6, and 8 hours of incubation, and no significant fungal death or increase was detected (percent survival range: 98.6–101.2). In contrast, conidia grown without melanin precursors subjected to terbinafine had a slightly decrease in cell viability. Fig 2 shows the percent survival of the strains exposed to terbinafine for 24 hours when compared to untreated control cells. It is particularly notable that pyomelanin significantly (P<0.05) protected four strains (Fig 2A) against the cidal effects of terbinafine. Moreover, some strains were able to increase their cell numbers even in the presence of two-times their MIC concentration (Table 1) for terbinafine. The presence of L-DOPA similarly enhanced the protection of cells against terbinafine, albeit growth did not occur at two-times MIC. The protection conferred by melanins produced by yeast cells grown under standard ambient conditions or 5%CO_2_ atmosphere was similar to that observed with conidia (Fig 2B).

### Tricyclazole did not directly interact with terbinafine

In order to assess whether tricyclazole directly interacted with terbinafine, we performed a checkerboard experiment with different terbinafine and tricyclazole concentrations. For all tested strains, the MICs for terbinafine did not change in presence of different concentrations of tricyclazole, resulting in a FICI = 1; thus showing a lack of interaction between these two drugs.

## Discussion

Melanins are ubiquitous pigment found in most pathogenic fungi, with biological functions related to fungal protection against harsh environmental and parasitic conditions [14]. One of the described functions for melanins produced by pathogenic fungi is the protection against antifungal drugs. This was observed for eumelanin producing fungi like *Cryptococccus neoformans* [22, 31], *Paracoccidioides brasiliensis* [32, 33], *Histoplasma capsulatum* [22], and *Penicillium marneffei* [34]. However, DHN-melanin producing strains of *Fonsecaea monophora* strains expressing melanin without precursors were not found to protect against antifungals [35] whereas DHN-melanin from *Madurella mycetomatis* and *Aspergillus fumigatus* were able to reduce the effects of ketoconazole and itraconazole [36].

Species from the *Sporothrix* complex produces at least three types of melanin [15–17]. The processes of melanin production in different *Sporothrix* species have not been fully elucidated, but, in general, *S*. *brasiliensis* produces DHN-melanin more rapidly and to a greater extent than *S*. *schenckii* [37]. Our results show that these two major species of the *Sporothrix* complex are able to produce these three types of melanin under the conditions described by the CLSI for the inoculum preparation, i.e. PDA medium at 37°C for seven days if the inducers are present. However, as detailed herein, production of eumelanin in RPMI-1640 medium could not be evaluated. As described by other authors [22, 34], the addition of L-DOPA to this medium rapidly resulted in L-DOPA autopolymerization. Moreover, inoculating the *Sporothrix* strains of our study in this mixture resulted in an impairment of growth for all strains in the presence or absence of antifungal drugs. A similar behavior was observed by other authors when growth of *M*. *mycetomatis* was attempted in a chemically defined minimal medium supplemented with L-DOPA [36]. However, the possibility that *Sporothrix* cells utilize some of the chemical compounds comprising RPMI-1640 medium for eumelanin synthesis cannot be excluded, since L-DOPA is not the sole precursor for this type of melanin [38]. Pyomelanin was also produced in the RPMI-1640 medium, though visible pigmentation was detectable only after nine days of growth, which is similar to what has been observed after incubating *Sporothrix* spp. in a minimal medium with 10mM L-tyrosine [17]. This fact complicated the evaluation of antifungal susceptibility during production of this pigment, since MIC readings must be performed at 48–52 hours [23]. We are aware that another limitation of the susceptibility testing protocol for *S*. *schenckii* is that it recommends testing the filamentous form of the fungus, and not the parasitic yeast form [24]. Moreover, only *S*. *schenckii* is included in CLSI M38-A2 protocol, and other *Sporothrix* species of clinical importance such as *S*. *brasiliensis* and *S*. *globosa* are not yet included [23]. This underscores the importance of additional studies to improve susceptibility testing of the genus *Sporothrix*.

We observed minor differences in susceptibility of melanized or non-melanized *Sporothrix* cells with the protocol for MIC determination described by the CLSI. This observation is consistent with that reported for other pathogenic fungi [22, 33, 34]. As previously described, the protection conferred by the *S*. *brasiliensis* pyomelanin against the killing effects of amphotericin B lasts for a few hours [17] and as the test is read after approximately 50 hours, we did not observe a protective effect in our model against amphotericin. However, tricyclazole reduced MIC readings of all strains to the lowest detectable value and the inhibitors sulcotrione and glyphosate in minor proportions, reduced MFC of the IPEC26449 *S*. *brasiliensis* strain. Moreover, the reduction in MIC values when the fungus grew in the presence of L-tyrosine, even within the intrinsic significant range of microdilution technique, suggests a role for pyomelanin and/or other metabolites from L-tyrosine catabolism in the resistance against antifungal drugs. Given that the microdilution assay is not an optimal tool for the observation of melanin antifungal protection, and these intrinsic limitations using this technique were noted, we also evaluated the effects of melanin in terbinafine susceptibility using a time-kill assay [22, 33].

The time-kill assay showed that terbinafine was fungicidal against *Sporothrix* strains producing only DHN-melanin and fungistatic when eumelanin or pyomelanin were produced before antifungal challenge. This phenomena was seen in *S*. *brasiliensis* and *S*. *schenckii*, despite the differences in the amount and time for melanin production by these two species [37]. *In vivo* production of melanin is described for several fungi [39–42] and it is very likely that a similar scenario occurs with the species of the *Sporothrix* complex, since sera from patients with sporotrichosis have antibodies with specificity to *Sporothrix* DHN and eumelanin [16]. Therefore, a preformed melanin can protect the fungus from terbinafine. It is important to note that melanin does not abolish the effects of terbinafine in our *in vitro* system, acting for a few hours after the contact between the fungus and the antifungal drug. In fact, terbinafine has been used in the treatment of sporotrichosis when itraconazole is contraindicated [10], with a similar efficacy between these two drugs [43].

The reduction of the MIC values observed for terbinafine in the presence of tricyclazole could be due to a chemical interaction between these two drugs, instead of a direct tricyclazole inhibitory action on DHN-melanin production. However, our checkerboard assay revealed no interaction between these two drugs, pointing that the mechanism of action of tricyclazole in terbinafine MIC reduction is based on its ability to inhibit melanin synthesis. Tricyclazole is indeed a potent inhibitor of DHN-melanin, decreasing production of pigment in *Sporothrix* even at low concentrations [16]. Terbinafine inhibits squalene epoxidase preventing ergosterol synthesis and affecting membrane and fungal cell wall structures [44]. As in the presence of tricyclazole there is no assembly of DHN-melanin at the fungal cell wall [16], terbinafine interference in fungal cell-wall synthesis could be enhanced, with a consequent hindering of fungal growth.

In conclusion, we report that melanins have the potential to protect strains of the major agents of sporotrichosis, *S*. *brasiliensis* and *S*. *schenckii*, against the antifungal effects of terbinafine. The potential protective role of melanins in the other *Sporothrix* species, such as *S*. *globosa*, *S*. *mexicana*, *S*. *luriei*, and *S*. *pallida* remains to be tested. To the best of our knowledge, this is the first report of a protective role of melanin-like pigments against this antifungal drug, suggesting that the development of new antifungal drugs targeting melanin synthesis could enhance treatment of this subcutaneous infection, potentially reducing durations of treatment and the administration of lower doses of currently prescribed antifungals. Further *in vivo* studies are required to address this hypothesis.
